# Electrospun Poly(butylene 2,5-furanoate) and Poly(pentamethylene 2,5-furanoate) Mats: Structure–Property Relationships and Thermo-Mechanical and Biological Characterization

**DOI:** 10.3390/molecules30040841

**Published:** 2025-02-12

**Authors:** Giulia Fredi, Sofia Santi, Michelina Soccio, Nadia Lotti, Andrea Dorigato

**Affiliations:** 1 Department of Industrial Engineering and INSTM Research Unit, University of Trento Via Sommarive 9, 38123 Trento, Italy; giulia.fredi@unitn.it; 2 Department of Civil, Chemical, Environmental, and Materials Engineering, University of Bologna, Via Terracini 28, 40131 Bologna, Italy; m.soccio@unibo.it (M.S.); nadia.lotti@unibo.it (N.L.); 3 Interdepartmental Center for Industrial Research on Advanced Applications in Mechanical Engineering and Materials Technology, CIRI-MAM, Viale del Risorgimento 2, 40136 Bologna, Italy; 4 Interdepartmental Center for Industrial Research on Buildings and Construction CIRI-EC, Via del Lazzaretto 15/5, 40131 Bologna, Italy; 5 Interdepartmental Center for Industrial Agro-Food Research, CIRI-AGRO, Via Quinto Bucci 336, 47521 Cesena, Italy

**Keywords:** electrospinning, biopolymers, furanoate polyesters, mechanical properties, drug delivery, biocompatibility

## Abstract

This study explores, for the first time, the application of electrospun biobased poly(butylene 2,5-furanoate) (PBF) and poly(pentamethylene 2,5-furanoate) (PPeF) mats in biomedical and drug delivery fields, through a careful investigation of their structure–property relationship. PBF mats, with a glass transition temperature (T_g_) of 25–30 °C and an as-spun crystallinity of 18.8%, maintained their fibrous structure (fiber diameter ~1.3 µm) and mechanical properties (stiffness ~100 MPa, strength ~4.5 MPa, strain at break ~200%) under treatment in physiological conditions (37 °C, pH 7.5). In contrast, PPeF mats, being amorphous with a T_g_ of 14 °C, underwent significant densification, with geometrical density increasing from 0.68 g/cm³ to 1.07 g/cm³, which depressed the specific (i.e., normalized by density) mechanical properties. DSC analysis revealed that the treatment promoted crystallization in PBF (reaching 45.9% crystallinity), while PPeF showed limited, but interestingly not negligible, structural reorganization. Both materials promoted good cell adhesion and were biocompatible, with lactate dehydrogenase release not exceeding 20% after 48 h. The potential of PBF mats for drug delivery was evaluated using dexamethasone. The mats exhibited a controlled drug release profile, with ~10% drug release in 4 h and ~50% in 20 h. This study demonstrates the versatility of these biopolyesters in biomedical applications and highlights the impact of polymer structure on material performance.

## 1. Introduction

The advances of controlled drug delivery systems, designed to release therapeutic agents within the body, represent a pivotal stride in modern medical research and pharmaceutical innovation. These systems tackle crucial challenges across various medical conditions, amplifying the efficacy and safety of medications while enhancing patient compliance and overall quality of life. Drug release mechanisms, including dissolution, osmosis, diffusion, and ion exchange, play integral roles, with diffusion and dissolution predominantly governing oral and transdermal routes [1]. Conventional drug delivery methods such as tablets, capsules, syrups, and ointments face limitations in bioavailability and blood drug level fluctuations, often failing to achieve sustained release [2]. Biomaterial-based drug delivery systems emerge as key players in regulating drug pharmacokinetics. Biomaterials, encompassing natural and synthetic biopolymers such as polysaccharides, proteins, lipids, peptides, and synthetic biopolyesters like polylactide, operate across a spectrum of sizes and administration routes. Crucially, biomaterials must align with formulation type, delivery site, and route, exhibiting characteristics such as biocompatibility, biodegradability, non-toxicity, hydrophilicity, muco-adhesiveness, and optimal mechanical strength. Nanocarriers, specifically those with sub-micron dimensions, exemplify enhanced drug loading and prolonged circulation, minimizing adverse effects [3].

In this context, nanofibrous drug delivery systems are gaining interest because of their high versatility. Their characteristics, including diameter, morphology, and porosity, can be finely tuned to achieve diverse drug release kinetics. These systems demonstrate high loading efficiency and precise spatial drug distribution. One way to produce such refined drug release systems is electrospinning [1,4,5]. Electrospun mats have been produced with a variety of materials, with natural (e.g., collagen, chitosan, gelatin) [6,7,8], petrochemical (e.g., polyethylene, polyvinyl alcohol, polyurethane, polyethylene oxide) [9], and biobased polymers (e.g., polylactide) [10] being prevalent.

Among biobased polymers stand poly(alkylene 2,5-furanoate)s (PAFs) [11]. Synthesized from 2,5-furandicarboxylic acid (FDCA) and glycols with varying alkyl chain lengths, PAFs exhibit superior thermal stability and thermo-mechanical and gas barrier properties compared to other petroleum-derived polyesters such as poly(alkylene terephthalate)s, thus starting to be considered as a more sustainable alternative for packaging applications [12,13,14]. Moreover, the presence of a polar furan ring enhances hydrophilicity and cell adhesion, akin to natural polymers [15]. In the PAF family, poly(ethylene 2,5-furandicarboxylate) (PEF) stands out as an extensively studied furan-based polyester [13,16,17], but other furanoate polyesters with longer alkyl chains have garnered attention for diverse applications, as their properties are largely dependent on the alkyl chain length [18,19,20]. For example, poly(butylene 2,5-furandicarboxylate) (PBF) and poly(pentamethylene 2,5-furandicarboxylate) (PPeF) are very similar from a structural point of view, as they differ only for one methylene group in the repeat unit’s alkyl chain, but they exhibit remarkably distinct physical, thermal, mechanical, and biological properties [21,22]. PBF is semicrystalline (melting temperature (T_m_) ~172 °C) and exhibits thermo-mechanical characteristics similar to poly(butylene terephthalate) (PBT), while PPeF is generally amorphous. This difference in the crystallization ability stems mainly from the so-called odd–even effect [18,23]. The C-odd-numbered glycolic subunit of PPeF decreases the glass transition temperature (T_g_) to ~17 °C compared to the ~35 °C of PBF and slows down the crystallization kinetics [18,24], which results in more ductile behavior with increased strain at break. Moreover, this remarkable difference in crystallization kinetics and T_g_ massively affects the aptitude of these two polymers to produce stable electrospun mats, with PPeF being considerably more challenging. Still, the feasibility of producing nanofibrous mats of PBF and PPeF by electrospinning was systematically investigated in a previous work of our group [21], which demonstrated the optimal conditions for the production of homogeneous PBF and PPeF electrospun mats with variable diameters. However, the mechanical properties of the produced PBF and PPeF mats and their potential as drug delivery systems are yet to be investigated.

Hence, this study aims to pioneer advanced drug delivery systems using electrospun PBF and PPeF mats. Through a comprehensive investigation of their biocompatibility and controlled release capability, this work seeks to unveil promising alternatives with potential treatment efficacy benefits. The work evaluates the biological properties of the produced mats via cell viability tests and drug release trials with dexamethasone, an anti-inflammatory drug, as the model molecule. Additionally, the study delves into the thermal and mechanical properties of PBF and PPeF electrospun mats and their stability under physiological conditions, highlighting the differences between the two polymers and providing a realistic simulation of their performance as implants in the human body or as transdermal patches for skin diseases.

## 2. Results and Discussion

The specimen names and labels are specified in Section 3, “Materials and Methods”, together with the meanings of the symbols and acronyms.

**Figure 1:**
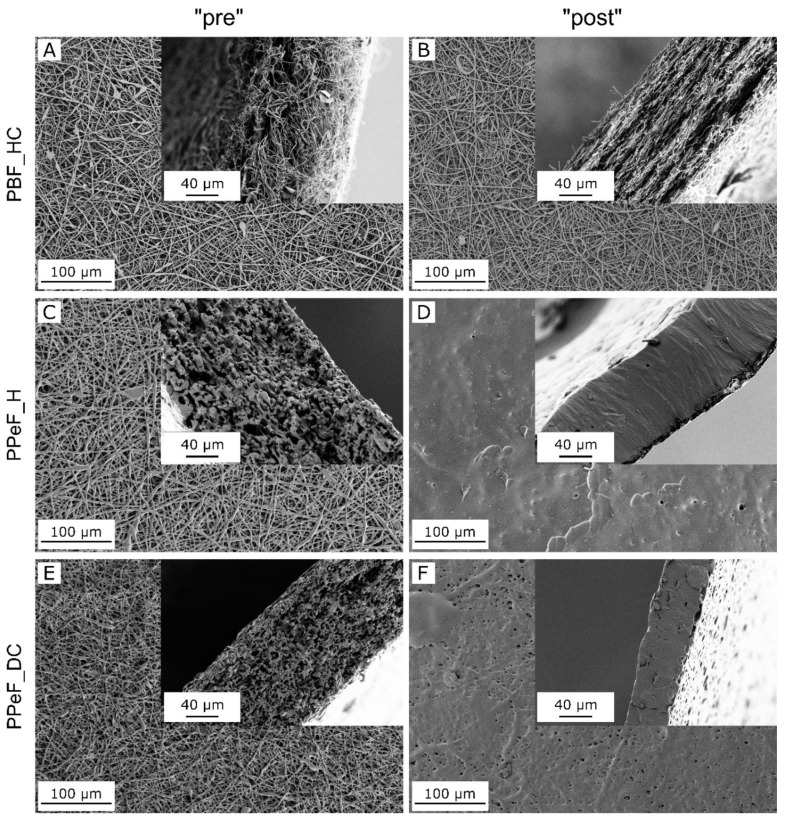
FESEM micrographs of PBF_HC (**A**,**B**), PPeF_H (**C**,**D**), and PPeF_DC (**E**,**F**) electrospun mats before (“pre”) (**A**,**C**,**E**) and after (“post”) (**B**,**D**,**F**) the treatment in physiological conditions at 37 °C for 24 h.

The microstructure of PBF and PPeF electrospun mats is highlighted by FESEM images reported in Figure 1. Striking are the morphological differences between the two polymers in terms of stability against the treatment at 37 °C. PBF mats show smooth and homogeneous fibers with a diameter of 1.3 ± 0.3 µm (Table 1) as well as a high porosity. These features are maintained after the treatment, as shown by the micrographs of the mats’ cross sections (inset in Figure 1A,B), the negligible increase in fiber diameter, and the minor variation in geometrical density and porosity (Table 1). This is positive as the retention of a fibrous and porous morphology is essential to allow gas and nutrient exchange for long-term cell survival. On the other hand, the treatment at 37 °C causes an almost complete loss of fibrous structure for the PPeF mats. In fact, PPeF_H_post and PPeF_DC_post show advanced densification, also highlighted by a geometrical density of 1.07 g/cm^3^ and 0.98 g/cm^3^, respectively, significantly higher than that measured in the “pre” state. It is worth mentioning that the density of the “pre” PPeF mats is already much higher than that of PBF_HC_pre and the porosity is lower, also qualitatively evident from the SEM images of the cross section of the electrospun mats (inset in Figure 1A,C,E), because PPeF mats are already characterized by partially interconnected and fused fibers. However, the main reason for the different behavior of complete densification of PPeF mats after the treatment in physiological conditions is the collapse of the fiber net above T_g_. This phenomenon is not present in PBF mats due to the higher T_g_ and the presence of the crystalline phase, as will be evident from the DSC results.

**Figure 2:**
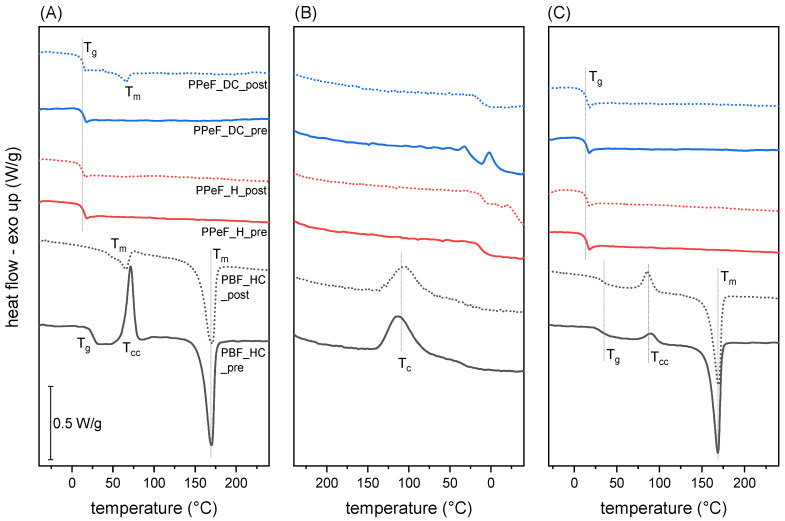
DSC thermograms of the prepared electrospun mats before (“pre”) and after (“post”) the treatment in physiological conditions: (**A**) first heating scan; (**B**) cooling scan; (**C**) second heating scan.

The main results of the DSC tests are reported in Figure 2 and Table 2. The DSC scans of PBF reveal its semicrystalline nature. In the first heating scan, PBF_HC_pre exhibits a distinct T_g_ at 25 °C, a pronounced cold crystallization peak at 72 °C, and a melting peak at 170 °C, unveiling a degree of crystallinity of 18.9%. The cooling scan of “pre” PBF highlights a crystallization at 113 °C and a distinct shoulder at 47 °C, likely due to a solid-state arrangement. The second heating scan shows a higher T_g_ (31 °C), likely due to the evaporation of moisture and residual solvent [25], followed by a cold crystallization peak at 90 °C and a melting peak at 173 °C. The DSC thermograms of PBF_HC_post reveal that the treatment at 37 °C for 24 h may not cause a loss of fibrous structure, as evidenced by SEM, but it certainly modifies the microstructural features and the crystallinity of the PBF itself. In the first heating scan, two endothermic peaks are evident, the first at 65 °C and the second at 170 °C, while no cold crystallization peak is observed. The treatment at 37 °C, above the PBF’s T_g_, promotes a strong crystallization of PBF. Considering only the enthalpy of the peak at 170 °C, the resulting degree of crystallinity is 45.9%, considerably higher than that of PBF_HC_pre. The endothermic peak at 68 °C does not resemble the typically sharp “enthalpic recovery” peak found in amorphous polymers aged slightly below their T_g_, but it may still be associated with the annealing of PBF occurring during the treatment in physiological conditions. A similar phenomenon was observed by Kainulainen et al. [26], who observed an additional endothermic peak at 84–95 °C after a thermal treatment at 75 °C for 300 h. This additional endothermic signal, combined with the considerable increase in crystallization, prevents the detection of the T_g_, which is why an “n.d.” is reported in Table 2. These differences between PBF_HC_pre and PBF_HC_post, caused by the treatment at 37 °C, are evened after the first melting event. In fact, the cooling scans of the two samples are very similar, and so are the second heating scans. More specifically, the similarities between the two T_g_s and the two T_m_s suggest that the treatment does not promote any degradation and molecular weight decrease in PBF. 

Conversely, PPeF_H_pre and PPeF_DC_pre are both fully amorphous, with a T_g_ at 14 °C. Indeed, the C-odd-numbered glycolic subunit affects the capacity of PPeF to crystallize, thereby explaining the amorphous microstructure with what is called the “odd–even effect” [27], while the relatively low T_g_ is explained by the presence of relatively long aliphatic subunits, which increase the molecular mobility. After the thermal treatment at 37 °C, PPeF_DC_post shows an endothermic peak at 67 °C, which may be associated with the development of a certain form of crystallinity due to a structural rearrangement of the polymer, similarly to what was observed by Martinez-Tong et al. [28] on PPeF aged at room temperature for 18 months. The reason why this phenomenon is not observed on PPeF_H is still unknown. However, this experiment confirms that the fully amorphous structure of as-spun PPeF mats is less stable at physiological temperatures than the semicrystalline morphology of PBF.

**Figure 3:**
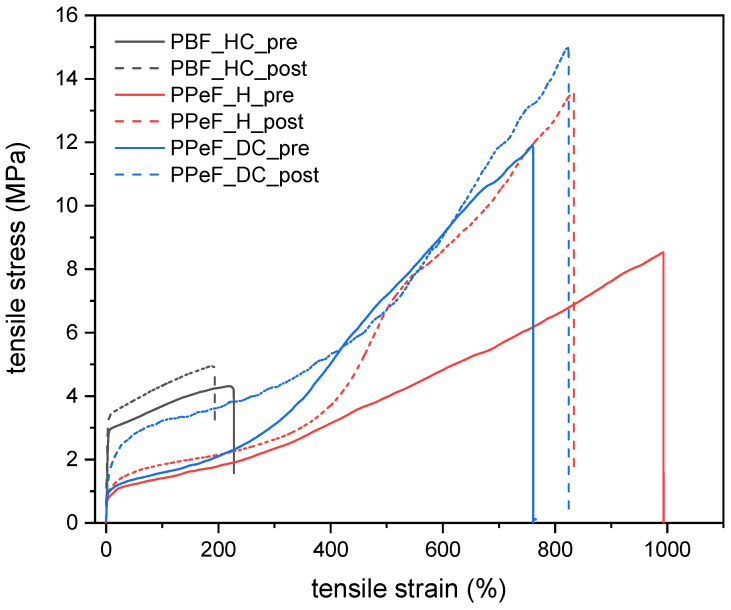
Representative stress–strain curves on the prepared electrospun mats before (“pre”) and after (“post”) the thermal treatment in physiological conditions.

The properties of the produced electrospun mats before and after the treatment in physiological conditions were also evaluated from a mechanical point of view. Figure 3 shows representative tensile stress–strain curves, while the most important results are summarized in Table 3. Considering that the mats have different densities, the values of the elastic modulus and ultimate tensile strength were also normalized to the geometric density (d) to facilitate the comparison of the results. The normalized modulus and strength are also reported in Table 3 as E/d and UTS/d.

The PBF-based mats exhibit a stress–strain curve characterized by a high initial slope until approx. 4 MPa, at which point the curves exhibit a marked knee with a reduced slope until failure. The load increases monotonically until failure, which makes the maximum stress and the stress at break coincide. The treatment in physiological conditions affects the properties only slightly, as PBF_HC_pre and PBF_HC_post show a stiffness of approx. 100 MPa, a strength of 4.5 MPa, and a strain at break of approx. 200%. The sample PBF_HC_post shows a slightly higher stiffness and strength and a slightly lower strain at break than the sample PBF_HC_pre, likely due to the slight densification and the noticeable increase in crystallinity accompanying the treatment.

In contrast, PPeF mats present more ductile behavior thanks to the amorphous nature of the polymer. This ductility allows them to undergo greater deformation without breaking; the mats before the thermal treatment, i.e., PPeF_DC_pre and PPeF_H_pre, exhibit an extended plastic region, with the stress increasing monotonically until breakage at very high strain levels (>700%), much higher than PBF_HC_pre. The maximum stress, also in this case equal to the stress at break, is also higher than that measured for PBF, reaching values up to 11.9 MPa, and the same is true for the initial stiffness. However, when density is considered, it can be noticed that the values of UTS/d and especially E/d of the PBF-based mats are higher than those of the PPeF-based ones, thanks to the very low density of the former. The treatment in physiological conditions promotes a further decrease in the normalized stiffness and strength, due to the considerable densification and the almost complete porosity occlusion measured for the PPeF-based mats.

Moreover, the variation in the stiffness of the prepared mats is a significant parameter for biomedical applications, Indeed, cells considerably modify their morphology, spreading, and adhesion to the substrate as a function of the stiffness of the substrate [29,30]. Although the optimal stiffness depends on the specific target application, the retention of the geometry and the mechanical properties in physiological conditions is generally desirable for the production of scaffolds for tissue regeneration or transdermal patches for drug delivery. Hence, PBF-based mats appear to be a more suitable candidate for this kind of application. It is also important to point out that skin-contact applications may require a lower operating temperature than the investigated temperature of 37 °C, which could also make the PPeF-based mats a good candidate for some specific applications.

**Figure 4:**
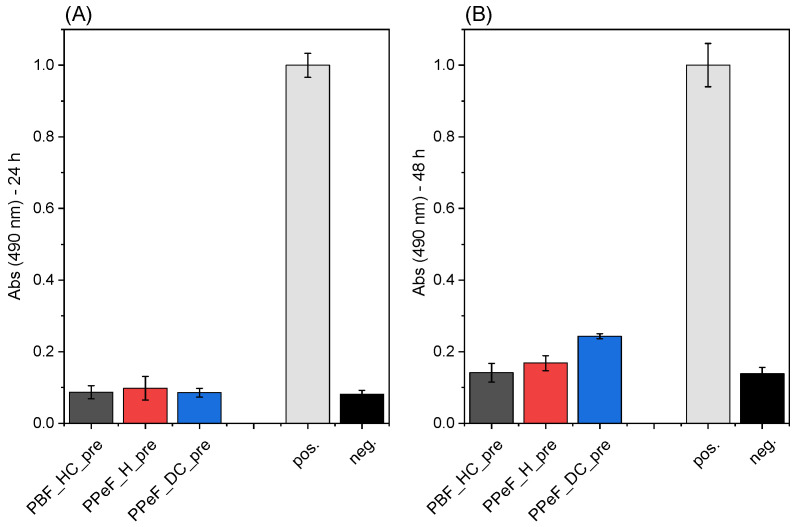
Results of LDH assay for PBF and PPeF electrospun mats after (**A**) 24 h and (**B**) 48 h. “pos” = positive control; “neg.” = negative control.

Hence, all the prepared PBF- and PPeF-based mats were subjected to preliminary biological tests by LDH assay to evaluate their potential cytotoxic effect. After 72 h of incubation, the culture medium conditioned by PBF or PPeF mats was put into contact with MRC5 cells, and the results are shown in Figure 4. According to the EN ISO 10993 standard [31], samples are considered cytotoxic when the LDH amount released into the medium is equal to or above 30% of the positive control. This condition is never reached for any of the tested samples, as the LDH released does not exceed 10% after 24 h and 20% after 48 h, which implies that none of the investigated mats exhibits cytotoxic effects on MRC5 cells.

**Figure 5:**
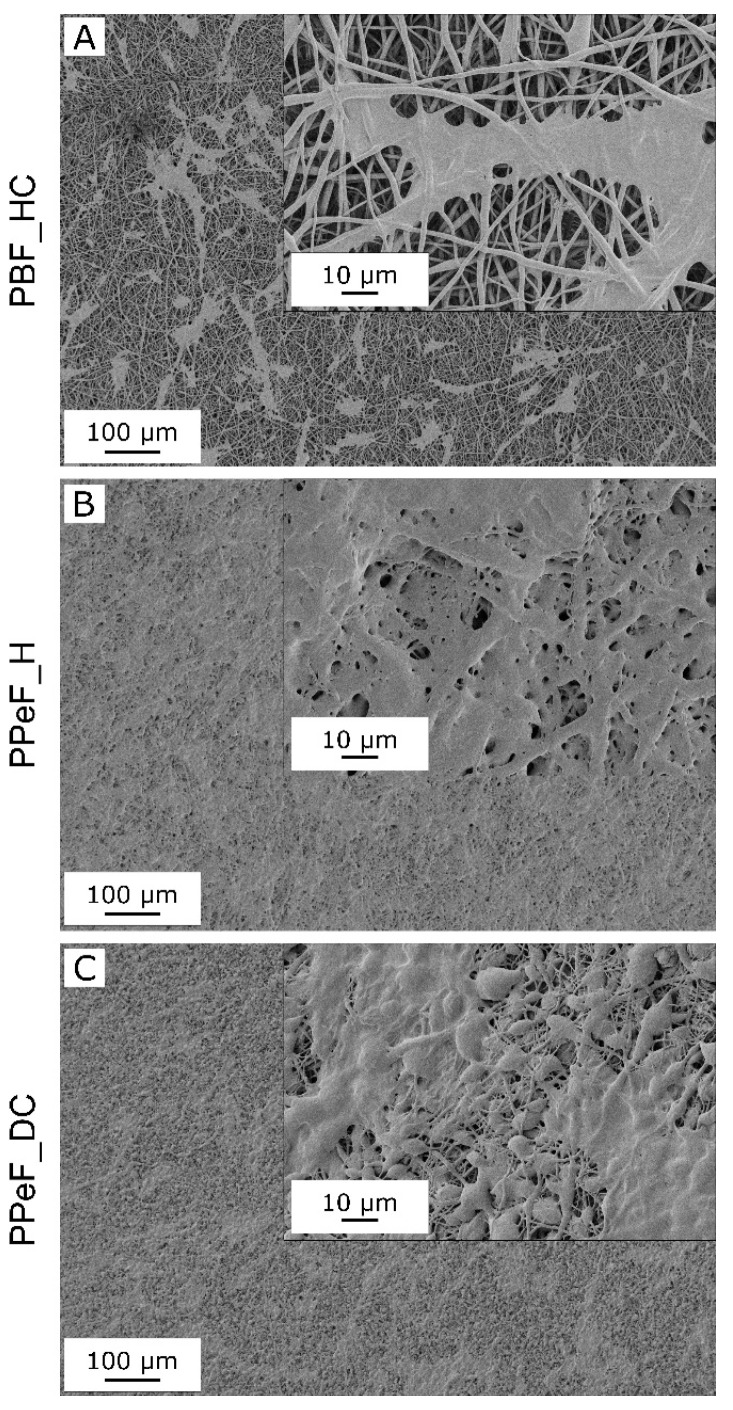
FESEM images of MRC5 adhesion on PBF and PPeF electrospun mats. (**A**) PBF_HC_pre, (**B**) PPeF_H_pre, and (**C**) PPeF_DC_pre.

Based on the cytotoxicity results, the same formulations were tested to study the preliminary cell adhesion at day 3, using the MRC5 cells as a cellular model considering applications such as skin wound healing [32]. The FESEM images reported in Figure 5 show good cell adhesion and long-shaped cytoskeletons of MRC5 on PBF_HC, PPeF_H, and PPeF_DC mats, which are thus suitable substrates for cell adhesion without any further surface functionalization. In the future, a more detailed analysis will be performed by confocal microscopy to appreciate the nuclei and the cytoskeletons of the cells and to highlight the density and the shape of the cell adhered.

**Figure 6:**
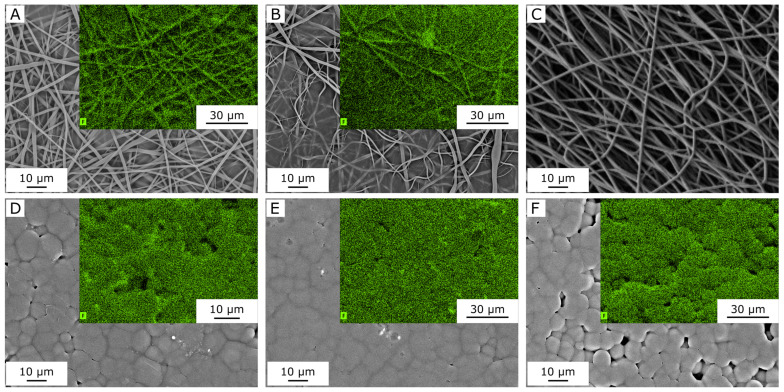
EDS-SEM images of electrospun PBF mats (**A**–**C**) and cast films (**D**–**F**) with or without DX. Insets represent the fluorine map. (**A**) PDMe-M; (**B**) PDCl-M; (**C**) P-M; (**D**) PDMe-C; (**E**) PDCl-C; (**F**) P-C. Insets are reported only for samples containing fluorine according to EDX analysis.

The promising results in terms of biocompatibility, cellular adhesion, and mechanical and structural stability of PBF mats encouraged the investigation of their performance as transdermal patches for drug delivery. The transdermal patch is generally used for the treatment of skin diseases, where the physiological conditions correspond to 25 °C and pH = 5.5 [33]. In these tests, PBF cast films loaded with dexamethasone (DX) dissolved in methanol or in chloroform (i.e., PDMe-C and PDCl-C samples, see Table 3) were compared to DX-loaded PBF electrospun mats (i.e., samples PDMe-M and PDCl-M). These samples differ from those analyzed so far because, in this case, the residual solvent was not removed by immersion in an ethanol solution, but they were simply dried in an oven to avoid early release of the drug. The comparison was performed in terms of DX loading capacity, by means of energy-dispersive X-ray spectroscopy (EDS) in combination with SEM micrography, as shown in Figure 6. The mass percentage (W%) and atom percentage (A%) values of carbon (C), oxygen (O), and fluorine (F) are reported in Table 4.

Analyzing the two DX-rich electrospun mats, PDMe-M (Figure 6A) reveals a fluorine (F) mass percentage of 1.21% and an F atom percentage of 0.8%. These values are higher than those observed in the PDCl-M (Figure 6B) samples, suggesting improved loading and distribution of the drug (DX) within the fibers. This indicates that DX dissolved in methanol disperses more uniformly in the PBF solution compared to DX dissolved in CHCl3/HFIP. Moreover, the F signal of cast films is generally higher than that of the electrospun mats, which could suggest a better loading of DX in the films. However, it is important to note that the unloaded mat, i.e., P-M (Figure 6C), shows no F signal, while the unloaded cast film, i.e., P-C (Figure 6F), contains an F content of approximately 1.6% by weight and 1.13% by atom count. This distinction indicates that the F signal detected in PDMe-M and PDCl-M samples originates solely from DX. In contrast, the F signal in PDMe-C and PDCl-C samples may also include residual HFIP from the solvent system. Based on all these findings, PDMe-M was identified as the most promising system for drug delivery.

**Figure 7:**
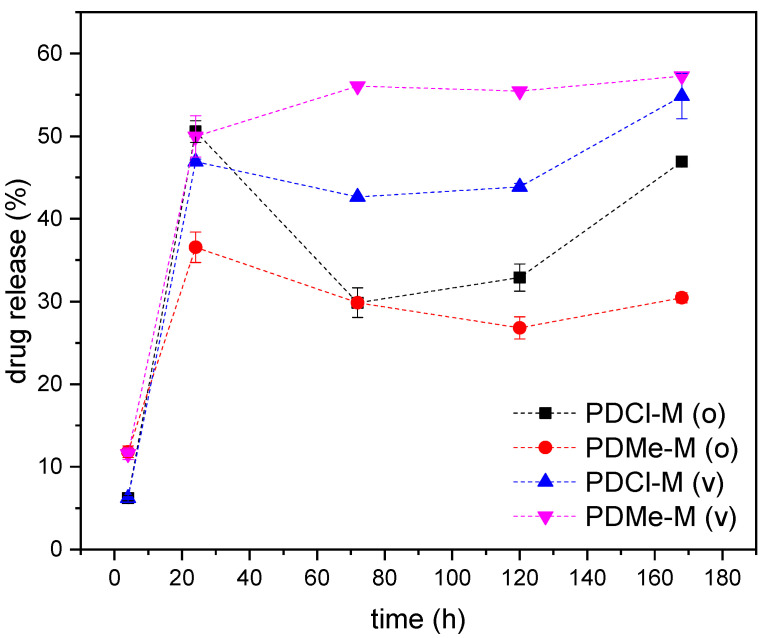
Results of drug release tests at 25 °C on PBF electrospun mats loaded with dexamethasone.

The release of dexamethasone (DX) from PBF electrospun mats at 25 °C is evaluated using UV-vis analysis, which also aids in determining the optimal drying method for residual solvent removal, i.e., oven drying at 60 °C for 24 h and vacuum drying at room temperature for 72 h. The DX release reported in Figure 7 is slower compared to other polymeric cast films [33] and electrospun mats [34,35,36] reported in the literature. A decrease in DX absorbance after 7 days of incubation under physiological conditions suggests drug modification or degradation, an important consideration for prolonged use in transdermal patches. The maximum drug release through PBF mats reaches 50%, indicating drug confinement that prevents complete release after 7 days of incubation. This may be due to the good affinity between PBF and DX and the difficulty for DX to move through a polymeric matrix below its T_g_. Both solvent removal treatments prove effective, with vacuum treatment leading to a slightly higher average drug release, although the profiles of the sample treated in the oven, and especially that of PDCl-M(o), do not monotonically increase, as would be expected from these types of tests.

To provide a more detailed evaluation of release kinetics, further analysis using high-performance liquid chromatography (HPLC) is recommended. This would allow for a more precise estimation of drug concentration released in the first 20 h, where additional data points are needed for a comprehensive assessment of the release profile. Moreover, further studies can focus on evaluating how release kinetics can be tuned by adjusting the mats’ porosity, permeability, and water capture properties, which is one of the advantages of using an electrospun mat instead of a cast film.

## 3. Materials and Methods

### 3.1. Materials

The materials used in this work are dimethyl 2,5-furandicarboxylate (2,5-DMF, Sarchem Labs, Farmingdale, NJ, USA, CAS 4282-32-0), 1,4-butanediol (1,4-BD, Sigma-Aldrich, St. Louis, MI, USA, 99% pur, CAS 110-63-4), 1,5-pentanediol (1,5-PD, Fluka, Honeywell International Inc., Charlotte, NC, USA, ~97% pur, CAS 111-29-5), hexafluoro-2-propanol, (HFIP, Carlo Erba, Cornaredo, Italy, CAS 920-66-1), chloroform (CHCl_3_, Carlo Erba, analytical standard, CAS 67-66-3, pur 99.5%,), dimethylformamide (DMF, Sigma-Aldrich, 99.5% pur, CAS 68-12-2,), and dexamethasone powder, suitable for cell culture (DX, Sigma-Aldrich, CAS 50-02-2, pur ≥ 97%).

### 3.2. Sample Preparation

**Figure 8:**
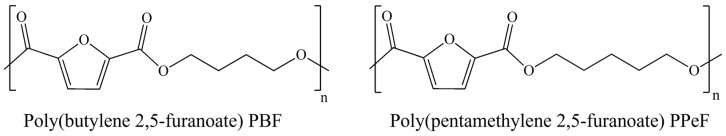
Chemical structures of PBF and PPeF.

**Synthesis of PBF and PPeF:** 2,5-DMF, 1,4-BD, and 1,5-PD were used to synthesize PBF and PPeF in a solvent-free polycondensation process as described in our previous work [37], using titanium tetrabutoxide (TBT) and titanium isopropoxide (TIP) as catalysts. The chemical structure of PBF (average molecular weight M_n_ = 36,500 g/mol) and PPeF (M_n_ = 43,900 g/mol) is reported in Figure 8.

**Figure 9:**
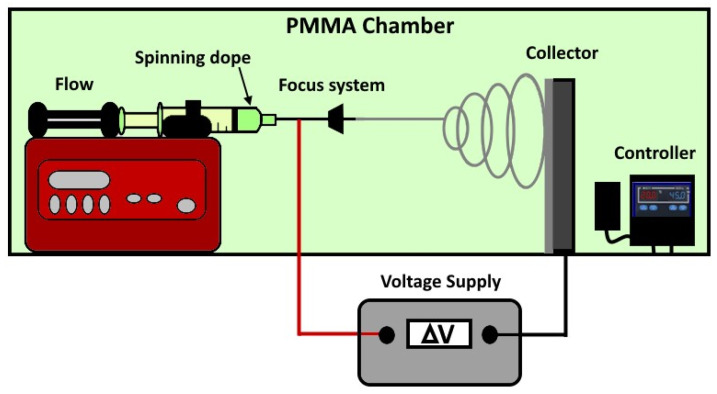
Schematic representation of the lab-scale electrospinning setup.

**Electrospinning of PBF and PPeF:** PBF and PPeF were solubilized in different solvent mixtures, employing HFIP, CHCl_3_, and DMF. The electrospinning setup (Figure 9) was confined in a poly(methylmethacrylate) (PMMA) chamber with monitored temperature and humidity of 20 °C and 45%, respectively. Electrostatic forces were generated by a DC voltage source of 24 kV, and the distance between the nozzle tip (diameter 0.9 mm) and the collector, a flat aluminum foil, was set at 15 cm. Different spinning rates were employed, ranging between 0.01 and 0.1 mL/min. PPeF and PBF mats with good microstructural quality were obtained under the optimized processing conditions described in our previous paper [21]. For PBF, the optimal processing conditions were obtained with a concentration of 0.11 g/mL in a 1:1 mixture of HFIP and CHCl_3_ and a flow rate of 0.01 mL/min. For PPeF, the optimal conditions were obtained either with a concentration of 0.2 g/mL in a 1:5 mixture of DMF and CHCl_3_ and a flow rate of 0.1 mL/min or with a concentration of 0.1 g/mL in HFIP and a flow rate of 0.05 mL/min. The optimized electrospinning parameters are reported in Table 5.

**Post-processing treatment:** All the prepared mats were washed in a 70% ethanol solution and vacuum-dried at room temperature for 12 h. This was performed on all samples to remove the residual solvent that could affect their thermal, mechanical, and biological performance and to assess the stability of the mats under a common sterilization procedure. Moreover, a treatment in physiological conditions (0.9% NaCl solution, pH 7.5, 37 °C, 24 h) was performed to evaluate their stability and their applicability as a biomedical device. Samples before this treatment were labeled “pre”, while those after treatment were labeled “post”. A list of the prepared electrospun mats with the applied post-processing parameters is summarized in Table 5.

### 3.3. Characterization

**Geometric density:** Disks with a diameter of 1 cm were die-cut for each sample and the thickness was measured with a micrometer to calculate the geometrical volume. The weight of each sample was also measured with a high-precision balance (0.001 mg). The average density of three specimens was measured per composition by dividing their mass by their geometrical volume. The total pore volume was determined as the difference between the material reference density and the calculated geometrical density divided by the material’s density. The material’s density was taken as the density of semicrystalline PBF, equal to 1.3 g/cm^3^ [38]. In fact, no data on the density of PPeF were found in the literature. The density of furandicarboxylate polyesters can vary as a function of the molecular structure, molar mass, and degree of crystallinity, and the density of amorphous PPeF is likely lower than that of the semicrystalline PBF, both because of its amorphous nature and due to the longer alkyl chain in the repeat unit; for example, the reported density of poly(ethylene furanoate) (PEF) varies in the range of 1.39–1.43 g/cm^3^ [39,40]. Hence, using the density of PBF as the material’s density for PPeF will likely slightly overestimate the porosity. Nevertheless, this procedure will result in an estimation of the porosity volume fraction in the prepared mats, which is a very important property for biomedical and drug release applications. 

**Microstructural characterization:** The microstructural features of the electrospun mats were investigated by a Zeiss Supra 60 (Carl Zeiss AG, Oberkochen, Germany) field emission microscope (FESEM), operating at an acceleration potential of 2.5–3.5 kV. Before the observations, the samples were sputtered (Quorum Q150V Plus, Laughton, UK) with a platinum–palladium coating for 20 s to render them conductive. The FESEM images were analyzed through the Image J^®^ software (v.1.53u) to determine the change in morphology before and after the treatment in physiological conditions.

**Thermal properties:** The thermal properties of the prepared electrospun mats were evaluated through differential scanning calorimetry (DSC), performed with a Mettler DSC 30 calorimeter (Mettler Toledo, Inc., Columbus, OH, USA). Approx. 4 mg of the mat was placed in an aluminum pan with a capacity of 40 µL and subjected to a heating/cooling/heating cycle at 10 °C/min between −50 °C and 250 °C, under a nitrogen flow of 100 mL/min. One specimen was tested for each composition. The glass transition temperature (T_g_) of PBF and PPeF was calculated as the midpoint of the glass-to-rubber transition inflection step. Moreover, the melting temperature (T_m_), the cold crystallization temperature (T_cc_), and the corresponding specific enthalpy values (ΔH_m_, ΔH_cc_) of PBF were determined. The crystallinity degree (χ) of PBF was calculated through Equation (1):

(1) $$\chi =\frac{\Delta H_{m}-{\Delta H}_{cc}}{{\Delta H}_{0}}\cdot 100$$

where ΔH_0_ is the theoretical melting enthalpy of fully crystalline PBF, equal to 129 J/g [41].

**Mechanical properties:** Quasi-static tensile tests were carried out on the prepared mats at room temperature and a relative humidity of 45% using an Instron^®^ (Norwood, MA, USA) 5969 universal testing machine equipped with a 100 N load cell. Specimens with a nominal width of 4 mm and thickness of 0.2 µm were cut out of the prepared mats with scissors, fixed on paper frames with a gauge length of 10 mm with scotch tape, and tested at a crosshead speed of 10 mm/min. At least six specimens were tested for each composition. The elastic modulus (E) was determined in correspondence with the maximum slope of the stress–strain curve, with the ultimate tensile strength (UTS) as the maximum stress divided by the load-bearing area (width × thickness) and the strain at break (ε_B_) as the elongation at break divided by the initial length.

**Biological evaluation:** The cytotoxicity of PBF_HC_pre, PPeF_H_pre, and PPeF_DC_pre mats was evaluated using a human lung fibroblasts cell line (MRC5), expanded and cultured under standard conditions (37 °C and 5% CO_2_) in minimal essential medium (MEM, Gibco, Thermofisher Scientific, Waltham, MA, USA) and 10% of inactivated fetal bovine serum (Euroclone, Pero, Italy), supplemented with 1% L-glutamine (Euroclone, Pero, Italy), sodium pyruvate (Gibco, Thermo Fisher Scientific, Waltham, MA, USA), non-essential amino acids (Sigma Aldrich, St. Louis, MO, USA), and antibiotic-antimycotic (Euroclone, Pero, Italy). All samples were sterilized in a 70% ethanol solution. An LDH cytotoxicity assay (Thermo Fisher Scientific) was performed to measure the amount of lactate dehydrogenase (LDH) released by cells during cell death. The test was performed following the EN ISO 10993 standard. Specifically, all samples without cells were incubated in a medium without phenol red and with heat-inactivated serum for 72 h (conditioned medium). After incubation, the conditioned medium was poured on MRC5 cells at 70% confluence, previously seeded at a density of 8000 cells/well in a 96-well plate and incubated for 48 h. Positive and negative controls were represented by fully lysate cells and cells cultured in a standard medium, respectively. After the incubation, all samples were prepared following the manufacturer’s instructions, and the LDH amount released in the medium was measured using a Tecan Infinite 200 microplate reader (Tecan Trading AG, Männedorf, Switzerland), recording the absorbance at 490 nm and the background at 680 nm. Five replicates were tested per sample.

Cell adhesion was also evaluated by FESEM images following the standard fixation protocol. Briefly, disks of 10 mm diameter obtained from the prepared mats were seeded with 20,000 cells/well, expanded, and cultured under standard conditions (37 °C and 5% CO_2_) in MEM for 3 days. Then, the mats were soaked in fixative 1 (glutaraldehyde 25% in cacodylic buffer 0.1 M (100 mL)) for 30 min at 4 °C. The samples were washed in fixative 2 (cacodylic buffer 0.1 M (100 mL)) three times and soaked in ethanol/water solution, increasing the ethanol content (30%, 50%, 70%, 90%, 100%) to dehydrate the cells. The samples were dried under a hood at room temperature before being sputtered with a platinum–palladium coating for 20 s to render them conductive and ready for FESEM analysis.

**Potential of PBF mats as transdermal patches for drug release:** Dexamethasone (DX), a corticosteroid used to treat many different inflammatory conditions such as allergic disorders and skin conditions, was used as the model drug. DX was solubilized in methanol at a concentration of 25 mg/mL as specified in the technical datasheet. DX was also solubilized at a concentration of 25 mg/mL in the 1:1 mixture of CHCl_3_:HFIP used to electrospin PBF. To produce DX-loaded electrospun mats, the two DX solutions were mixed with the PBF solution to reach the final concentration of 0.05 mg DX per ml solution. The solutions were then electrospun to obtain DX-rich mats. The two solvent systems in which DX was solubilized (i.e., methanol and 1:1 mixture of CHCl_3_:HFIP) were compared to check whether methanol, the ideal solvent for DX according to the technical datasheet, affected the electrospinning of PBF. These tests were only performed on PBF, as PPeF mats presented morphological instability in physiological conditions.

The spinning dopes were also cast in glass Petri dishes to produce PBF/DX films; 1 mL of the resulting solutions was cast in a glass Petri dish with a diameter of 3.5 cm. The films were compared with the electrospun mats to appreciate the confinement of the drug into the fibers. Electrospun and cast samples without DX were also prepared as controls. All samples were post-treated either in an oven at 60 °C for 24 h or under vacuum for 72 h. These treatments were selected after the failure of a preliminary trial for the solvent removal performed under vacuum for 24 h, which did not allow complete solvent removal. No ethanol solution was used to post-treat the DX-loaded samples to prevent early release of the drug.

The drug release kinetics were measured by a spectrophotometer at Abs of 244 nm after incubation of a 10 mg PBF mat for 4, 24, 72, 120, and 168 h at 25 °C in 1 mL of buffer solution at pH 5.5, which are the physiological conditions of the skin surface. Given the initial concentration of DX and PBF in the initial spinning dope (0.05 mg/mL and 0.11 g/mL, respectively), the maximum DX concentration in 1 mL buffer solution could be calculated as 0.00454 mg/mL, which would have indicated a total release of the drug. The experimental DX concentration (M) after the incubation time was measured via UV-vis spectrophotometry, and the measured absorbance values at 244 nm were converted in DX concentrations through a calibration curve. The calibration curve was determined by analyzing a series of DX solutions of known concentration, ranging from 0.001 to 0.1 mg/mL and determining the height of the max peak at 244 nm. Hence, the data of concentration could be converted into percentual release by knowing the maximum nominal DX release, i.e., 0.00454 mg/mL, for the specimens evaluated in this work. The drug distribution in the produced samples was also observed by energy-dispersive X-ray spectroscopy (EDS) (Carl Zeiss AG, Oberkochen, Germany) after gold sputtering, highlighting the presence of fluorine belonging to DX. The test was performed on all electrospun mats and cast films dried at 60 °C for 24 h. Table 6 summarizes all the samples characterized by UV-Vis and EDS-SEM analysis.

## 4. Conclusions

Based on the comprehensive investigation of electrospun poly(butylene 2,5-furanoate) (PBF) and poly(pentamethylene 2,5-furanoate) (PPeF) mats, several important conclusions can be drawn regarding their potential as drug delivery systems. This study revealed significant differences in the structural stability and mechanical properties of PBF and PPeF mats under physiological conditions at 37 °C. PBF mats demonstrated superior stability, maintaining their fibrous and porous structure after treatment at 37 °C, while PPeF mats underwent substantial densification. This difference is attributed to the higher glass transition temperature and semicrystalline nature of PBF, in contrast to the amorphous structure of PPeF, as revealed by DSC.

Mechanical testing further highlighted the advantages of PBF mats, which exhibited consistent properties before and after physiological treatment. PBF mats maintained a stiffness of approximately 100 MPa, a strength of 4.5 MPa, and a strain at break of approx. 200% both before and after treatment. In contrast, PPeF mats showed more pronounced changes in mechanical behavior post-treatment, with their normalized (i.e., divided by density) stiffness and strength decreasing due to considerable densification and porosity occlusion.

Biological evaluations yielded promising results for both PBF and PPeF mats. Neither material exhibited cytotoxic effects on MRC5 cells, with LDH release not exceeding 20% after 48 h. Both demonstrated good cell adhesion properties without the need for surface functionalization. These findings suggest potential applications in biomedical fields such as tissue engineering or wound healing.

The investigation of PBF mats as transdermal drug delivery systems using dexamethasone as a model drug provided valuable insights. Electrospun mats allowed for effective drug loading, with dexamethasone dissolved in methanol showing superior dispersion within the PBF fibers compared to other solvent systems. However, the drug release tests also revealed some limitations. The release kinetics of dexamethasone from PBF mats were slower compared to literature reports on other polymeric systems, with a drug release plateau of a maximum of 50%. This slow release rate may be suboptimal for applications requiring rapid drug delivery. Additionally, a decrease in dexamethasone absorbance after 7 days of incubation suggested potential drug degradation, which could limit the long-term efficacy of the delivery system.

In conclusion, this research demonstrates the promising potential of PBF electrospun mats for biomedical applications, particularly in the realm of drug delivery systems. The combination of structural stability, consistent mechanical properties, and biocompatibility positions PBF mats as a worthy candidate for further development in this field. However, the slow drug release kinetics and potential drug degradation over time highlight areas for improvement. Future research directions should focus on optimizing the drug release profile, enhancing long-term drug stability, and exploring the performance of these mats with a wider range of therapeutic agents.

## Figures and Tables

**Table 1:** Geometric density and fiber diameter (mean ± standard deviation) of PBF and PPeF electrospun mats.

Sample	Geometric Density (g/cm^3^)	Porosity (vol%)	Fiber Diameter (µm)
PBF_HC_pre	0.27 ± 0.01	79 ± 1	1.3 ± 0.3
PBF_HC_post	0.29 ± 0.01	78 ± 1	1.4 ± 0.4
PPeF_H_pre	0.68 ± 0.11	48 ± 8	2.5 ± 0.4 *
PPeF_H_post	1.07 ± 0.07	18 ± 5	-
PPeF_DC_pre	0.74 ± 0.03	43 ± 2	2.0 ± 0.5 *
PPeF_DC_post	0.98 ± 0.04	25 ± 3	-

* Fibers are not perfectly circular and partially fused.

**Table 2:** Results of DSC tests on “PBF_HC, PPeF_H, and PPeF_DC mats, “pre” and “post” treatment in physiological conditions.

		PBF_HC_pre	PBF_HC_post	PPeF_H_pre	PPeF_H_post	PPeF_DC_pre	PPeF_DC_post
H1	T_g_ (°C)	25	n.d.	14	12	14	13
	T_cc_ (°C)	72	-	-	-	-	-
	ΔH_cc_ (J/g)	32.0	-	-	-	-	-
	T_m_ (°C)	170	65/170	-	-	-	67
	ΔH_m_ (J/g)	56.4	12.7/59.2	-	-	-	3.7
	χ (%)	18.9	45.9	-	-	-	-
C	T_c_ (°C)	113	104	-	-	-	-
	ΔH_c_ (J/g)	47.7	33.7	-	-	-	-
H2	T_g_ (°C)	31	32	13	13	13	14
	T_cc_ (°C)	90	86	-	-	-	-
	ΔH_cc_ (J/g)	5.3	8.3	-	-	-	-
	T_m_ (°C)	173	169	-	-	-	-
	ΔHm (J/g)	50.0	44.5	-	-	-	-
	χ (%)	34.6	28.0	-	-	-	-

H1 = first heating scan; C = cooling scan; H2 = second heating scan.

**Table 3:** Results of quasi-static tensile tests on the prepared electrospun mats.

Samples	E (MPa)	E/d MPa/(g/cm^3^)	UTS (MPa)	UTS/d MPa/(g/cm^3^)	ε_b_ (%)
PBF_HC_pre	96 ± 6	352 ± 24	4.3 ± 0.3	15.7 ± 1.5	225 ± 19
PBF_HC_post	105 ± 19	359 ± 65	5.1 ± 0.2	17.2 ± 0.8	189 ± 12
PPeF_H_pre	136 ± 3	199 ± 5	7.9 ± 1.5	11.5 ± 2.2	894 ± 119
PPeF_H_post	127 ± 16	119 ± 15	12.5 ± 2.5	11.6 ± 2.3	789 ± 105
PPeF_DC_pre	111 ± 19	151 ± 26	11.9 ± 1.5	16.1 ± 2.1	772 ± 71
PPeF_DC_post	140 ± 16	142 ± 16	14.8 ± 2.5	14.9 ± 2.9	614 ± 117

E = elastic modulus; UTS = ultimate tensile strength; ε_b_ = strain at break; d = density.

**Table 4:** Mass percentage (W%) and atom percentage (A%) of carbon (C), oxygen (O), and fluorine (F) present in PBF electrospun mats and cast samples, obtained through EDS-SEM images.

Samples	C		O		F	
	W%	A%	W%	A%	W%	A%
PDMe-M	65.13 ± 0.36	71.45 ± 0.33	33.67 ± 0.37	27.72 ± 0.33	1.21 ± 0.03	0.84 ± 0.02
PDMe-C	61.93 ± 0.30	68.62 ± 0.27	35.96 ± 0.33	29.91 ± 0.29	2.10 ± 0.06	1.47 ± 0.04
PDCl-M	67.13 ± 0.51	73.17 ± 0.47	32.34 ± 0.63	26.47 ± 0.55	0.52 ± 0.15	0.36 ± 0.10
PDCl-C	62.93 ± 0.30	67.62 ± 0.27	36.96 ± 0.33	28.91 ± 0.29	2.30 ± 0.06	1.27 ± 0.04
P-M	62.45 ± 0.13	68.90 ± 0.11	37.55 ± 0.13	31.10 ± 0.11	-	-
P-C	61.21 ± 0.13	67.91 ± 0.11	37.17 ± 0.23	30.95 ± 0.18	1.62 ± 0.23	1.13 ± 0.16

**Table 5:** List of the prepared PBF and PPeF non-woven mats with the applied processing and post-processing parameters. After electrospinning, all samples were treated in a 70% ethanol solution and vacuum-dried at room temperature for 12 h.

Label	Polymer	Conc. (g/mL)	Solvent	Flow Rate (mL/min)	Post-Processing in Physiological Sol. at 37 °C
PBF_HC_pre	PBF	0.11	1:1 HFIP/CHCl_3_	0.01	No
PBF_HC_post	PBF	0.11	1:1 HFIP/CHCl_3_	0.01	Yes
PPeF_H_pre	PPeF	0.1	HFIP	0.10	No
PPeF_DC_pre		0.2	1:5 DMF/CHCl_3_	0.05	
PPeF_H_post	PPeF	0.1	HFIP	0.10	Yes
PPeF_DC_post		0.2	1:5 DMF/CHCl_3_	0.05	

**Table 6:** Summary of PBF samples characterized by UV-Vis and EDS-SEM analysis for drug release tests.

Label	DX Solvent	Sample Form	Post-Treatment	UV-Vis	EDS-SEM
P-M(o)	-	Electrospun Mat	60 °C 24 h	-	✓
PDMe-M(o)	Methanol	Electrospun Mat	60 °C 24 h	✓	✓
PDCl-M(o)	1:1 CHCl_3_:HFIP	Electrospun Mat	60 °C 24 h	✓	✓
PDMe-M(v)	Methanol	Electrospun Mat	Vacuum 72 h	✓	-
PDCl-M(v)	1:1 CHCl_3_:HFIP	Electrospun Mat	Vacuum 72 h	✓	-
P-C(o)	-	Cast film	60 °C 24 h	-	✓
PDMe-C(o)	Methanol	Cast film	60 °C 24 h	-	✓
PDCl-C(o)	1:1 CHCl_3_:HFIP	Cast film	60 °C 24 h	-	✓

P = PBF; DMe = dexamethasone dissolved in methanol; DCl = dexamethasone dissolved in chloroform; M = electrospun mat; C = cast film; (o) = oven-dried at 60 °C; (v) = vacuum-dried at room temperature.

## Data Availability

Data will be made available on request.
